# Tapetum-Dependent Male Meiosis Progression in Plants: Increasing Evidence Emerges

**DOI:** 10.3389/fpls.2019.01667

**Published:** 2020-01-16

**Authors:** Xiaoning Lei, Bing Liu

**Affiliations:** ^1^School of Public Health, Key Lab of Public Health Safety of the Ministry of Education and NHC Key Laboratory of Health Technology Assessment, Fudan University, Shanghai, China; ^2^Hubei Provincial Key Laboratory for Protection and Application of Special Plants in Wuling Area of China, College of Life Sciences, South-Central University for Nationalities, Wuhan, China; ^3^Key Laboratory for Biotechnology of the State Ethnic Affairs Commission, College of Life Sciences, South-Central University for Nationalities, Wuhan, China

**Keywords:** male meiosis, tapetal cell specification, tapetum PCD, gene expression, sRNAs

## Abstract

In higher plants, male meiosis is a key process during microsporogenesis and is crucial for male fertility and seed set. Meiosis involves a highly dynamic organization of chromosomes and cytoskeleton and specifically takes place within sexual cells. However, studies in multiple plant species have suggested that the normal development of tapetum, the somatic cell layer surrounding the developing male meiocytes, is indispensable for the completion of the male meiotic cell cycle. Disrupted tapetum development causes alterations in the expression of a large range of genes involved in male reproduction. Moreover, recent experiments suggest that small RNAs (sRNAs) present in the anthers, including microRNAs (miRNAs) and phased, secondary, small interfering RNAs (phasiRNAs), play a potential but important role in controlling male meiosis, either by influencing the expression of meiotic genes in the meiocytes or through other unclear mechanisms, supporting the hypothesis that male meiosis is non-cell autonomously regulated. In this mini review, we summarize the recorded meiotic defects that occur in plants with defective tapetum development in both Arabidopsis and crops. Thereafter, we outline the latest understanding on the molecular mechanisms that potentially underpin the tapetum-dependent regulation of male meiosis, and we especially discuss the regulatory role of sRNAs. At the end, we propose several outstanding questions that should be addressed in future studies.

## Introduction

Male meiosis is a specialized type of cell division that gives rise to daughter cells with a reduced chromosome number. It is therefore particularly important for the production of viable spores, plant fertility, and the consistence of plant ploidy over generations (Bhatt et al., 2001; De Storme and Geelen, 2013). For detailed cellular processes and the genetic control of male meiosis in plants, we refer to excellent reviews (Mercier et al., 2015; Wang and Copenhaver, 2018). Many genes are preferentially expressed during male meiosis, indicating that the male meiotic cell cycle is genetically regulated by a complex network (Cnudde et al., 2006; Crismani et al., 2006; Chen et al., 2010; Deveshwar et al., 2011; Yang et al., 2011; Dukowic-Schulze et al., 2014; Florez-Zapata et al., 2014). Dysfunction of meiosis-related genes may either lead to unbalanced chromosome segregation with consequent aneuploid progenies and impaired male fertility, or, alternatively, it may cause meiotic restitution, a non-reductional meiotic event resulting in the production of unreduced gametes (Ferdous et al., 2012; Andreuzza et al., 2015; Yuan et al., 2018; Yang et al., 2019a; Yang et al., 2019b; Zhang et al., 2019). Additionally, male meiosis is also controlled by epigenetic modifications at both the DNA and protein levels (Choi et al., 2018; Osman et al., 2018; Underwood et al., 2018; Walker et al., 2018; Yuan et al., 2018; Yang et al., 2019b).

Tapetum is the innermost cell layer in the anther, which surrounds the developing pollen mother cells (PMCs) and/or microspores supplying nutrition and enzymes required for microsporogenesis and pollen maturation. The differentiation and development processes of anther tissues, including tapetum and sporogenous cells, which give rise to PMCs, have been comprehensively reviewed (Scott et al., 2004). Generally, the development of tapetum can be classified as having three stages: tapetum specification, tapetal cell binucleation, and degeneration through programmed cell death (PCD) (Scott et al., 2004). In Arabidopsis, the fate of anther somatic cells and sexual cells is determined at ﬂoral stage 8/anther stage 4, and both the tapetum and PMCs are formed by ﬂoral stage 9/anther stage 5 (Sanders et al., 1999). Defective tapetum development is often associated with the disrupted development of meiocytes and/or pollen and reduced/impaired fertility (Ji et al., 2013; Cao et al., 2015; Yi et al., 2016; Chen et al., 2018). The development of tapetum and PMCs is coordinately regulated, and it is believed they partially share some regulatory factors (Yang et al., 1999). It becomes clearer that male meiosis is not merely regulated by the expression of genes within the meiocytes, but also involves the participation of molecules generated by surrounding somatic cells, especially the tapetum. Here, we have outlined the impact of disrupted tapetum development on male meiosis; moreover, we have discussed the underlining mechanisms, especially for the role of small RNAs in tapetum-dependent meiosis control.

## Completion of the Male Meiotic Cell Cycle Relies on Normal Tapetum Development

### The Specification of Tapetum is Vital for Meiosis Progression and Maturation

An arrested meiotic cell cycle and/or failed meiocyte maturation can both occur in plants with disrupted tapetum development at early anther stages (Murmu et al., 2010; Cui et al., 2018). In Arabidopsis, the specification of the tapetal cell layer at early anther stages is predominantly controlled by the Leu-rich repeat receptor protein kinase (LRR-RLK) *Excess Microsporocytes 1* (*EMS1*)/*Extra Sporogenous Cells* (*EXS*) and its ligand *Tapetum Determinant 1* (*TPD1*), two determinants for the cell fate of tapetum precursors and the maintenance of tapetal cells (Zhao et al., 2002; Yang et al., 2003; Yang et al., 2005; Huang et al., 2016). The corresponding null mutant plants exhibit aborted development of male meiocytes prior to the occurrence of meiotic cytokinesis (Zhao et al., 2002; Yang et al., 2003). The activity of EMS1/EXS depends on its autophosphorylation status (Jia et al., 2008), which is enhanced by two LRR-RLKs: Somatic Embryogenesis Receptor-Like Kinase1 (SERK1) and SERK2 (Li et al., 2017b). A downstream factor of EMS1/EXS, β-carbonic anhydrases (βCAs), was recently identified; it is phosphorylated by EMS1/EXS (Huang et al., 2017). Tetrads cannot be formed in both the *βca1 βca2 βca4* and the *βca1 βca2 βca3 βca4* mutants (Huang et al., 2017). Remarkably, BRI1 EMS Suppressor 1 (BES1) family members, including BES1, Brassinazole Resistant 1 (BZR1), BES1/BZR1 Homolog 1 (BEH1), BEH2, BEH3, and BEH4, as key transcription factors in brassinosteroid (BR) signaling, regulate tapetum PCD by acting downstream of the EMS1-TPD1-SERK1/2 module but independently of the BR signaling (Yin et al., 2005; Chen et al., 2019). The quintuple *bes1-1 bzr1-1 beh1-1 beh3-1 beh4-1* (*qui-1*) mutant is defective for the cell fate determination of tapetum and exhibits arrested tetrad formation (Chen et al., 2019).

Similar observations have been recorded in crops. Multiple Sporocyte 1 (MSP1), the orthologous protein to AtEXS/AtEMS1, controls the number of sporocytes in rice. Dysfunction of MSP1 results in an impaired tapetum layer initiation with a resultant abnormal number of PMCs. Male meiosis in *msp1* is aborted prior to the completion of prophase I (Nonomura et al., 2003). MSP1 interacts with its ligand, OsTDL1A (TPD1-like 1A), and both the *ostdl1a* single and the *ostdl1a msp1* double mutants phenocopy *msp1*, showing disrupted tapetum formation, and male meiosis is arrested at late prophase I (Yang et al., 2016). These findings indicate that a regular specification of tapetal cells is required for meiosis progression. A basic helix-loop-helix (bHLH) protein, TDR Interacting Protein2 (TIP2), regulates the initiation and development of tapetum in rice by modulating the transcription of Tapetum Degeneration Retardation (TDR) and bHLH transcription factor Eternal Tapetum1 (EAT1), two positive regulators of the PCD of tapetum (Li et al., 2006; Zhang et al., 2008; Niu et al., 2013; Fu et al., 2014). Male meiosis is normally initiated in the *tip2* mutant, but the meiocytes cannot maturate into anaphase I (Fu et al., 2014). *Defective Tapetum and Meiocytes 1* (*DTM1*), which encodes for an endoplasmic reticulum (ER) membrane protein specifically existing in cereals, regulates early stage tapetum development (Yi et al., 2012). The null *dtm1* mutant is defective for initial tapetum differentiation and later degeneration, and male meiosis is arrested at prophase I (Yi et al., 2012), indicating that the normal function of ER is crucial for the development of tapetum and male meiocytes. At the same time, in maize, *Multiple Archesporial Cells 1* (*MAC1*), which encodes for an ortholog of rice TDL1A, regulates cell proliferation prior to tapetum specification at early anther stages (Wang et al., 2012). Null mutation of *MAC1* leads to an excess of archesporial cells and causes a disordered periclinal division with associated failed tapetum formation (Wang et al., 2012). The development of male meiocytes in *mac1* is mostly arrested at metaphase I with the rest aborted before metaphase II (Wang et al., 2012).

In Arabidopsis, the post-specification development of tapetum is predominantly controlled by a module composed of Dysfunctional Tapetum 1 (DYT1), Defective in Tapetal Development and Function 1 (TDF1), Aborted Microspores (AMS), Male Sterile 188 (MS188), and MS1 (Yang et al., 2007; Zhang et al., 2007; Gu et al., 2014; Ferguson et al., 2017; Li et al., 2017a; Lou et al., 2018). This regulatory pathway regulates the expression of a large number of genes encoding for lipid transfer proteins, E3 ubiquitin ligases, metacaspases and cysteine protease; these are required for tapetum PCD, tapetum wall degradation, and pollen wall formation (Ito et al., 2007; Ma et al., 2012; Li et al., 2017a). The homologs of the corresponding proteins in this module have been identified in crops (Li et al., 2006; Zhang et al., 2010; Li et al., 2011; Cai et al., 2015; Guo et al., 2018; Yang et al., 2019c), and this is indicative of the conservation of their roles among species. For detailed information of the genetic regulation of tapetum development we refer to a recent review (Verma, 2019). At early anther stages, the bHLH transcription factor DYT1 regulates tapetum development by modulating the expression of downstream transcription regulators and the factors required for lipid metabolism and pollen wall formation (Feng et al., 2012). DYT1 acts downstream of EMS1/EXS and the *dyt1* mutant displays a phenotype similar to *ems1*/*exs*, i.e. arrested male meiosis progression without the occurrence of cytokinesis (Zhang et al., 2006), hinting that EMS1/EXS may regulate meiosis progression through DYT1. Moreover, the expression of DYT1 is largely suppressed in the BES1 family quintuple *qui-1* mutant, hinting that EMS1/EXS may regulate DYT1 through the EMS1-TPD1-SERK1/2-BES1 pathway (Chen et al., 2019). Whether the other members of the DYT1-TDF1-AMS-MS188-MS1 module are involved in controlling meiosis remains unknow. In rice, the transcription factor Undeveloped Tapetum 1 (UDT1) regulates tapetum development at an early meiosis stage, and the mutant displays abnormal tapetum morphology during meiosis (Jung et al., 2005). The male meiocytes in *udt1* have normal chromosome dynamics, but dyads cannot develop into the tetrad stage, suggesting that the *udt1* mutation-induced tapetum lesions primarily impact meiosis II. These findings indicate that a normal formation of the tapetum cell layer and its development at early anther stages are of essential importance for the undergoing and maturation of male meiosis.

### PCD of Tapetal Cells is Required for Meiotic Cytokinesis and Tetrad Formation

At late microsporogenesis stages, tapetal cells undergo PCD and the precise occurrence of this process is important for tapetum degeneration and pollen development (especially for the pollen wall formation) (Sun et al., 2018b; Bai et al., 2019; Gao et al., 2019; Mondol et al., 2019; Shukla et al., 2019; Xu et al., 2019; Yang et al., 2019c; Yang et al., 2019d; Zheng et al., 2019). In both Arabidopsis and crops, *GAMYB* genes regulate tapetum PCD in a microRNA-controlled manner and are believed to act under the control of gibberellic acid (GA) signaling (Kaneko et al., 2004; Millar and Gubler, 2005; Allen et al., 2007; Guo and Ho, 2008; Alonso-Peral et al., 2010; Liu et al., 2010). In Arabidopsis, MYB33 and MYB65 redundantly regulate the PCD of tapetal cells, with the double *myb33 myb65* mutant exhibiting enlargement of tapetum at meiosis stages (Millar and Gubler, 2005). The expression of *MYB33* and *MYB65* is not dramatically influenced in the *dyt1* and the *ams* mutants, and there is no interaction between DTY1 and MYB33 (Zhang et al., 2006; Feng et al., 2012; Ma et al., 2012). The expression studies suggest that the GA-MYBs and the DYT1-AMS signaling pathways may act in parallel in controlling tapetum development, but it is not known yet whether these two signaling pathways coordinately function during tapetum development and, if they do, how they do so.

In Arabidopsis, DELLA family members, Repressor of *ga1-3* (RGA) and GA Insensitive (GAI), and GAMYB-like proteins, MYB33 and MYB65, are key GA-signaling repressors and downstream responsive factors, respectively (Peng et al., 1997; Silverstone et al., 1997; Silverstone et al., 1998; Millar and Gubler, 2005). Loss of function of both the mutant alleles (the double *rga-24 gai-t6* and *myb33 myb65* mutants) leads to defective male meiotic cytokinesis and restituted unreduced gametes, suggesting a potential link between the GA-DELLA-MYB signaling-dependent tapetum PCD and the completion of male meiosis (Plackett et al., 2014; Liu et al., 2017). In rice, the bHLH142 protein interacts with TDR, forming a complex that coordinately regulates the expression of EAT1 (Niu et al., 2013; Ko et al., 2014). The *bHLH142* T-DNA insertion mutant *ms142* exhibits defective tapetum PCD and retarded meiotic progression, and the male meiocytes cannot develop into tetrads (Ko et al., 2014). A PHD domain transcription protein, TDR Interacting Protein 3 (TIP3), was recently reported, and it plays a role in tapetum PCD and pollen development by regulating the expression of genes required for tapetum layer initiation and degeneration, including *MSP1*, *GAMYB*, *UDT1*, *TDR*, and *EAT1* (Yang et al., 2019d). The *tip3* mutant displays delayed tapetum PCD but undergoes normal male meiosis (Yang et al., 2019d). It could be the case that, by binding with TDR, TIP3 activates the expression of downstream targets specifically related to tapetum PCD and pollen wall formation (Yang et al., 2019d). The association of defective tapetum PCD and irregular chromosome dynamics during meiosis progression is also recorded in the rice *eat1* mutant. Moreover, in tomatoes, the loss of function of Male Sterile 10^35^ (MS10^35^) brings about defects in tapetum PCD, and male meiocytes in the mutant cannot develop into tetrads (Jeong et al., 2014). These reports suggest the notion that the meiotic cell cycle and meiocyte development are at least partly interconnected with normal tapetum PCD.

**Figure 1:**
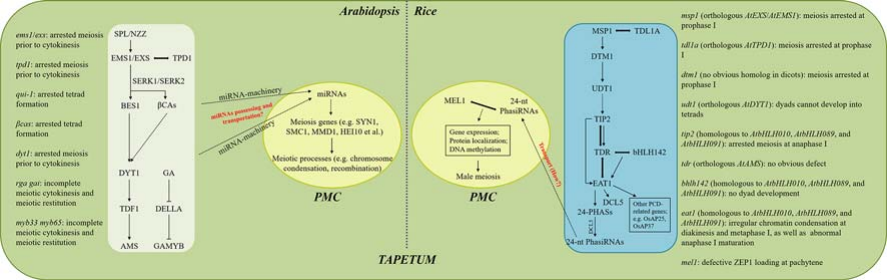
Proposed modeling of tapetum-dependent regulation of male meiosis in Arabidopsis and rice. Meiotic phenotypes in plants with different tapetum defects in Arabidopsis and rice are summarized on the left and right, respectively. The signaling pathways occurring in the white and blue boxes take place in the tapetal cells, while the ones shown in the central yellow ovals are happening in the PMCs. Arrows indicate the positive regulation of gene expression or the occurring of events, while the inhibiting lines represent negative regulation. Double-headed arrows indicate protein interaction. Dotted arrows indicate unclear or uncertain mechanisms. Key but uncertain mechanisms are marked by red.

The meiosis defects in the tapetum mutants in Arabidopsis and rice have been summarized (**Figure 1**). It seems that Arabidopsis and rice have different sensitivities to the influenced tapetum development in the context of male meiosis. In Arabidopsis, alterations occurring either during tapetal cell differentiation or later degeneration stages are likely to cause irregularities in meiotic cytokinesis, and early tapetum development seems more important (**Figure 1**). In contrast, more programs during rice meiosis, including completion of prophase I, chromosome condensation at diakinesis and metaphase I, and maturation of anaphase I, are sensitive to influenced tapetum, and defects at early tapetum stages tend to influence earlier meiosis processes (**Figure 1**). These differences suggest that the tapetum-dependent regulation of male meiosis may be varied among species, probably due to distinct regulatory molecules. Moreover, since genes with a role in regulating tapetum differentiation also regulate downstream factors required for tapetum PCD; e.g. *TIP2* in rice (Fu et al., 2014), and the corresponding mutants display meiosis defects at specific developmental stages, it is likely that the control of male meiosis is coordinated with the progression of tapetum development.

On the other hand, since there is no obvious evidence showing that irregular tapetum causes univalent formation at diakinesis stage, it is likely that tapetum development is not essentially required for chromosome dynamics at prophase I. However, whether altered tapetum development would induce changes in the landscape of meiotic recombination is not known yet. It is thus interesting to examine whether several main events during early prophase I, e.g. double strand break (DSB) formation and distribution, length of axis and synaptonemal complex (SC), and genome DNA methylation, which are the determinants of the landscape of meiotic recombination (Armstrong et al., 2002; Higgins et al., 2005; Wang et al., 2011; Barakate et al., 2014; Yelina et al., 2015; He et al., 2017; Chambon et al., 2018; Choi et al., 2018; Underwood et al., 2018; Walker et al., 2018; Xue et al., 2018), are affected by the dysfunction of tapetum.

## Molecular Mechanisms Underpinning Tapetum-Dependent Regulation of Male Meiosis

### Dysfunction of Tapetum Influences the Expression of Meiosis-Related Genes

Although a constitutively activated GA-DELLA signaling interferes with male meiotic cytokinesis in Arabidopsis, the DELLA protein RGA is not active in the developing meiocytes but is instead highly active in the tapetum (Plackett et al., 2014; Liu et al., 2017), suggesting that cell-to-cell communication exists between the two different cell types in regulating male meiosis. One piece of the evidence of this is that Sporocyteless (SPL), which plays an essential role in controlling differentiation of anther layers (including the tapetum) and the formation of sporogenous cells, is specifically expressed in the sporogenous cells and the sporocytes (Yang et al., 1999), indicating that, at early anther stages, the development of anther somatic tissues relies on a functional signaling from the sexual cells. In maize, the expression of Argonaute (AGO) protein AGO18b increases during tassel development and peaks at meiosis stages. AGO18b is highly expressed in both the tapetum and developing meiocytes (Zhai et al., 2014), and its repression was found to interfere with sister chromatids segregating during meiosis II (Sun et al., 2018c). This indicates that tapetum and male meiocytes have overlapping transcriptome profiles, and many genes with a key role in either tapetum development or male meiosis, or both, are expressed in both cell types (Caryl et al., 2000; Grelon et al., 2001; Armstrong et al., 2002; Schommer et al., 2003; Millar and Gubler, 2005; Chen et al., 2010; Yang et al., 2011; Yi et al., 2012; Lu et al., 2014). The activity of meiotic regulators relies on normal tapetum development. In Arabidopsis, the expression of *DUET*/*MMD1*, a chromatin regulator required for multiple meiotic processes, including chromatin condensation, spindle organization, and meiotic cytokinesis, is reduced in the *ems1*/*exs* mutant (Ma et al., 2012; Andreuzza et al., 2015; Wang et al., 2016). In addition, the expression of genes involved in cytoskeleton organization is decreased in Arabidopsis *ams* plants (Xu et al., 2010). Similarly, in rice, the transcripts of genes related to the cell cycle or male meiosis, including *Pair1*, are reduced in the *dtm1* and *udt1* mutants (Jung et al., 2005; Yi et al., 2012). At the same time, the activity of Pollen Semi-Sterility 1 (PSS1), a microtubule-stimulated ATPase required for microtubule organization during rice meiosis, is diminished in the *dtm1* mutant (Zhou et al., 2011; Yi et al., 2012). The *pss1* mutant shows delayed chromosome movement at both anaphase I and II, and synchronous chromosome segregation occurs at the end of meiosis II in *pss1*, resulting in meiotic-restitution and triads (Zhou et al., 2011). Notably, disorganized microtubular configurations (e.g. destabilized, curved, and/or omitted microtubule bundles) in the *pss1* meiocytes mimic that of the Arabidopsis *rga-24 gai-t6* and the *myb33 myb65* plants (Zhou et al., 2011; Liu et al., 2017). This phenotypic similarity hints that GA-interfered meiotic cytokinesis may be due to an influenced activity of microtubule-related proteins, but whether this is a secondary effect of irregular GA signal-induced tapetum defects is not clear (Plackett et al., 2011). Collectively, it seems that tapetal cells get involved in regulating male meiosis at least partially by affecting the expression of meiosis genes, with the communicating mechanism between the two cell types unknown.

### Small RNAs in Anther Tissues, Including Tapetum, Play a Potential but Important Role in Regulating Male Meiosis

In plants, small RNAs (sRNAs) largely exist in male reproductive tissues and regulate developmental processes. The landscape of sRNAs was recently evidenced to correlate with meiotic gene expression or DSB distribution in Arabidopsis (Huang et al., 2019a). Based on different functional patterns, sRNAs can mainly be classified as small interfering RNAs (siRNAs) and microRNAs (miRNAs), which directly target DNA and silence gene expression by impacting DNA methylation through the RNA-directed DNA methylation (RdDM) mechanism or by post-transcriptionally regulating gene expression, respectively, reviewed by (Axtell, 2013; Borges and Martienssen, 2015; Kamthan et al., 2015). The miRNA miR159 is able to regulate male reproductive development by suppressing downstream transcription factors MYB33 and MYB65 (Alonso-Peral et al., 2010). It is plausible that miRNA regulates male meiosis by targeting its downstream meiosis genes. Actually, in multiple plant species, miRNAs have been proposed to target genes related to male meiosis (Omidvar et al., 2015; Dukowic-Schulze et al., 2016). For example, in wheat, *CCR4-associated factor 1* (*CAF1*), which is involved in meiotic progression, is targeted by miR2275 (Sun et al., 2018a); in sorghum, miR171 targets *RPA1c*, which is required for meiotic DNA repair (Chang et al., 2009; Dhaka et al., 2019). In Arabidopsis, HYL1, HEN1, DCL1, HST, and AGO1 are miRNA-machinery components required for miRNA maturation ((Park et al., 2002; Reinhart et al., 2002; Baumberger and Baulcombe, 2005; Hiraguri et al., 2005; Park et al., 2005; Yu et al., 2005), and reviewed by (Chen, 2008)). Dysfunction of these miRNA-processing factors leads to varied levels of alteration in different male meiosis events: chromatin condensation, chiasma formation, and recombination (Oliver et al., 2017). An expression study found that genes involved in meiotic chromatin organization (e.g. *SYN1* and *SMC1*) in the *hst-21* mutant and the genes related to homologous recombination (e.g. *SPO11-1*, *DMC1*, *RAD1*, and *MSH4*) in the *dcl1-9* mutant are differentially expressed compared to wild-type Arabidopsis (Oliver et al., 2017). It is thus possible that meiosis-related genes are regulated by miRNAs processed by this machinery. However, the mechanism of miRNA-dependent regulation of male meiosis awaits further elucidation, and the exact role of tapetum is not yet clear.

Phased siRNAs (phasiRNAs, 21 or 24 nucleotides long) are plant-specific siRNAs and have gained increasing focus due to their conservation in plants and their putative role in plant development control [(Kakrana et al., 2018; Xia et al., 2019), reviewed by (Fei et al., 2013)]. The 21-nt and 24-nt phasiRNAs are synthesized through miR2118- and miR2275-dependent pathways, respectively, and are preferentially produced in the male reproductive tissues (Song et al., 2012). In maize, AGO18b binds miR2275 and both 21- and 24-nt phasiRNAs (Sun et al., 2019), and the *ago18b* mutant exhibits affected sister chromatid segregation (Sun et al., 2018c). Similarly, rice phasiRNA-binding AGO protein MEL1 is required for a homologous synapsis with the mutant displaying defective ZEP1 loading on pachytene chromosomes (Komiya et al., 2014). Most MEL1-binding phasiRNAs have been clarified to be 21nt phasiRNAs, which are synthesized through miR2118- and Dicer Like 4 (DCL4)-dependent processing pathways from large intergenic non-coding RNAs (lincRNAs) (Liu et al., 2007; Song et al., 2012; Komiya et al., 2014). At the same time, in rice, the tapetal cell nucleus-localized EAT1 coordinately acting with UDT1 positively regulates the expression of precursor RNAs of 24-nt phasiRNAs (24-PHASs) at 101 genome loci in the anthers (Ono et al., 2018). By binding with other bHLH factors, EAT1 activates the expression of DICER-LIKE5 (DCL5), a processor of double-stranded 24-PHASs (Ono et al., 2018). TIP2 is involved in these processes by a similar manner as EAT1 (Ono et al., 2018). The *eat1* mutant shows defects in both tapetum PCD, irregular chromatin condensation at diakinesis and metaphase I, as well as abnormal anaphase I maturation (Ono et al., 2018). Moreover, in the *msp1* and *ostdl1a* mutants, miR2275 and 24-nt phasiRNAs, as well as their precursor PHAS mRNAs, are decreased (Fei et al., 2016). These findings suggest phasiRNAs have a putative role in male meiosis, and indicate that tapetum-located proteins with a key role in regulating tapetum development are able to modulate phasiRNA abundance by influencing their biosynthesis, which subsequently affects meiosis directly or indirectly.

EAT1-dependent 24-nt phasiRNAs bind MEL1 that is specifically expressed in meiocytes (Nonomura et al., 2007; Komiya et al., 2014; Ono et al., 2018), suggesting that phasiRNAs are transported from tapetal cells to PMCs. Sequencing analysis of small RNAs from maize mutants defective for either different anther somatic lobe layers or meiocytes has been applied to uncover the metabolism and dynamics of meiotic phasiRNAs in male reproductive tissues (Zhai et al., 2015). In maize anthers, 24-nt meiotic phasiRNAs are detectable in both tapetum and the developing meiocytes; however, the phasiRNA biosynthesis factor RNA-dependent RNA polymerase 6 (RDR6) was found to be specifically expressed in the tapetum (Zhai et al., 2015). Similarly, maize DCL5, which is specifically required for 24-nt phasiRNA precursor processing, displays a much higher expression level in the tapetum than meiocytes. The *dcl5* mutant has significantly reduced mature 24-nt phasiRNAs and has a defect in tapetal cell binucleation (Teng et al., 2018). These facts hint that male reproductive phasiRNAs are produced in the tapetum (Huang et al., 2019b) and transported into PMCs to undertake their function. The mechanism controlling dynamics of phasiRNAs between the tapetum and developing meiocytes has not been found yet. A newly developed sRNA-FISH tool can be applied to understand the ‘synthesis-movement-function’ fashion of phasiRNAs between the cell types (Huang et al., 2019b).

Interestingly, in maize, the accumulation of 21-nt phasiRNAs is more coincident with pre-meiotic anther development events, while the 24-nt phasiRNAs reach their peak during meiosis I (Zhai et al., 2015). On the contrary, in rice anthers, 21-nt phasiRNAs peak during meiosis progression, but the 24-nt phasiRNAs are more abundant when meiosis is about to finish (Fei et al., 2016; Tamim et al., 2018). In cotton, the abundance of 24-nt phasiRNAs peak at the tetrad stage (Xia et al., 2019). Although the miR2275-dependent 24-nt phasiRNA biogenesis mechanism is highly conserved in many organs of eudicots, it is absent in Arabidopsis anthers (Zheng et al., 2015; Xia et al., 2019). The difference of the phasiRNA accumulation pattern suggests that the putative phasiRNA-dependent regulation of male meiosis is different among different grasses. Given that phasiRNAs might be somehow involved in tapetum-dependent regulation of male meiosis, we speculate that the different profiles and patterns of phasiRNAs could be the cause of varied sensitivity of meiosis programs between Arabidopsis and rice to the tapetum defects. In any case, other sRNAs, particularly miRNAs might be responsible for the tapetum-dependent male meiosis control in Arabidopsis (Huang et al., 2019a).

How phasiRNAs function in meiosis regulation is not clear. Prediction of reproductive phasiRNA targets has been attempted in rice, but more investigation, especially of the molecular and biochemical evidence, is required (Patel et al., 2018). Reduced ZEP1 foci on the *mel1* pachytene chromosomes suggests that either the expression or localization of meiosis proteins depends on the successful binding of phasiRNAs with AGO proteins (Komiya et al., 2014). In maize, 21-nt and 24-nt phasiRNAs are able to increase methylation levels at genome CHH contexts in meiocytes (Dukowic-Schulze et al., 2016). phasiRNAs may thus influence meiosis by modulating DNA methylation and chromatin remodeling. Whether phasiRNA induces DNA methylation by interacting with RNA-directed DNA methylation components, such as AGO4 (Qi et al., 2006), or through other pathways needs further investigation.

## Discussion

Male meiosis is crucial for the production of viable haploid gametes and the male fertility of higher plants. Many findings suggest that the male meiotic cell cycle is not merely regulated by the machineries and signaling within the sexual cells but is instead non-cell autonomously controlled by the surrounding somatic tissues, especially for tapetum. Although many advances have been made in the understanding of molecular factors involved in the control of male meiosis with the genetic network being increasingly and comprehensively shaped, the mechanism of the small RNA-mediated regulation of meiotic programs is not fully understood. Recent studies in crops are starting to reveal how small RNAs, especially miRNAs and phasiRNAs, may play roles in mediating the impact of tapetum on the development and maturation of male meiocytes. Phenotypic observations suggest that tapetum-dependent male meiosis in different plant species may be regulated by different molecular mechanisms and may be based on different types of small RNAs. With an aim for the molecular breeding of crops by manipulating male meiotic cell division, it is of great importance to understand how male meiosis is influenced by the tapetum. Meanwhile, considering the fact that the development of tapetum is hypersensitive to environmental conditions, uncovering the genetic basis underlining tapetum-dependent male meiosis is crucial for the breeding of stress-tolerant crops. Finally, we propose several outstanding questions that should be addressed in future studies:

1. Does tapetum development have an impact on the landscape of male meiotic recombination and, if so, how?
2. What molecular factors in the tapetum or other somatic tissues within the anther regulate the activity of small RNAs, and how do they coordinately regulate male meiosis?
3. How many and what kind of small RNAs are involved in the regulation of male meiosis, and how are they processed and transported?
4. Does tapetum development have an impact on the epigenetic modifications of the genome DNA or the meiosis-related factors in the developing male meiocytes?
5. Does tapetum mediate the impact of environmental stresses on male meiosis and, if so, how?

Based on increasingly developed molecular and cytological tools, we believe that these questions can be answered in the near future.

## Author Contributions

XL drafted the manuscript. BL designed, edited, and conceived the manuscript. The authors have read and approved the manuscript.

## Funding

The work is supported by University Research Foundation Project 304/YZZ18007 (to BL).

## Conflict of Interest

The authors declare that the research was conducted in the absence of any commercial or financial relationships that could be construed as a potential conflict of interest.

The reviewer YW declared a shared affiliation, with no collaboration, with one of the authors, XL, to the handling editor at the time of the review.

## References

[B1] Allen R. S. Li J. Stahle M. I. Dubroue A. Gubler F. Millar A. A. (2007). Genetic analysis reveals functional redundancy and the major target genes of the Arabidopsis miR159 family. Proc. Natl. Acad. Sci. U.S.A. 104, 16371–16376. 10.1073/pnas.0707653104 17916625 PMC2042213

[B2] Alonso-Peral M. M. Li J. Li Y. Allen R. S. Schnippenkoetter W. Ohms S. (2010). The microRNA159-regulated GAMYB-like genes inhibit growth and promote programmed cell death in Arabidopsis. Plant Physiol. 154, 757–771. 10.1104/pp.110.160630 20699403 PMC2949021

[B3] Andreuzza S. Nishal B. Singh A. Siddiqi I. (2015). The chromatin protein DUET/MMD1 controls expression of the meiotic gene TDM1 during male Meiosis in Arabidopsis. PloS Genet. 11, e1005396. 10.1371/journal.pgen.1005396 26348709 PMC4562639

[B4] Armstrong S. J. Caryl A. P. Jones G. H. Franklin F. C. (2002). Asy1, a protein required for meiotic chromosome synapsis, localizes to axis-associated chromatin in Arabidopsis and Brassica. J. Cell Sci. 115, 3645–3655. 10.1242/jcs.00048 12186950

[B5] Axtell M. J. (2013). Classification and comparison of small RNAs from plants. Annu. Rev. Plant Biol. 64, 137–159. 10.1146/annurev-arplant-050312-120043 23330790

[B6] Bai W. Wang P. Hong J. Kong W. Xiao Y. Yu X. (2019). Earlier Degraded Tapetum1 (EDT1) encodes an ATP-citrate lyase required for tapetum programmed cell death. Plant Physiol. 181 (3), 1223–1238. 10.1104/pp.19.00202 31515447 PMC6836821

[B7] Barakate A. Higgins J. D. Vivera S. Stephens J. Perry R. M. Ramsay L. (2014). The synaptonemal complex protein ZYP1 is required for imposition of meiotic crossovers in barley. Plant Cell 26, 729–740. 10.1105/tpc.113.121269 24563202 PMC3967036

[B8] Baumberger N. Baulcombe D. C. (2005). Arabidopsis ARGONAUTE1 is an RNA Slicer that selectively recruits microRNAs and short interfering RNAs. Proc. Natl. Acad. Sci. U.S.A. 102, 11928–11933. 10.1073/pnas.0505461102 16081530 PMC1182554

[B9] Bhatt A. M. Canales C. Dickinson H. G. (2001). Plant meiosis: the means to 1N. Trends Plant Sci. 6, 114–121. 10.1016/s1360-1385(00)01861-6 11239610

[B10] Borges F. Martienssen R. A. (2015). The expanding world of small RNAs in plants. Nat. Rev. Mol. Cell Biol. 16, 727–741. 10.1038/nrm4085 26530390 PMC4948178

[B11] Cai C.-F. Zhu J. Lou Y. Guo Z.-L. Xiong S.-X. Wang K. (2015). The functional analysis of OsTDF1 reveals a conserved genetic pathway for tapetal development between rice and Arabidopsis. Sci. Bull. 60, 1073–1082. 10.1007/s11434-015-0810-3

[B12] Cao H. Li X. Wang Z. Ding M. Sun Y. Dong F. (2015). Histone H2B monoubiquitination mediated by HISTONE MONOUBIQUITINATION1 and HISTONE MONOUBIQUITINATION2 is involved in anther development by regulating tapetum degradation-related genes in rice. Plant Physiol. 168, 1389–1405. 10.1104/pp.114.256578 26143250 PMC4528728

[B13] Caryl A. P. Armstrong S. J. Jones G. H. Franklin F. C. (2000). A homologue of the yeast HOP1 gene is inactivated in the Arabidopsis meiotic mutant asy1. Chromosoma 109, 62–71. 10.1007/s004120050413 10855496

[B14] Chambon A. West A. Vezon D. Horlow C. De Muyt A. Chelysheva L. (2018). Identification of ASYNAPTIC4, a component of the meiotic chromosome axis. Plant Physiol. 178, 233–246. 10.1104/pp.17.01725 30002256 PMC6130017

[B15] Chang Y. Gong L. Yuan W. Li X. Chen G. Li X. (2009). Replication protein A (RPA1a) is required for meiotic and somatic DNA repair but is dispensable for DNA replication and homologous recombination in rice. Plant Physiol. 151, 2162–2173. 10.1104/pp.109.142877 19812186 PMC2785997

[B16] Chen C. Farmer A. D. Langley R. J. Mudge J. Crow J. A. May G. D. (2010). Meiosis-specific gene discovery in plants: RNA-Seq applied to isolated Arabidopsis male meiocytes. BMC Plant Biol. 10, 280. 10.1186/1471-2229-10-280 21167045 PMC3018465

[B17] Chen Z. S. Liu X. F. Wang D. H. Chen R. Zhang X. L. Xu Z. H. (2018). Transcription factor OsTGA10 is a target of the MADS protein OsMADS8 and is required for tapetum development. Plant Physiol. 176, 819–835. 10.1104/pp.17.01419 29158333 PMC5761795

[B18] Chen W. Lv M. Wang Y. Wang P. A. Cui Y. Li M. (2019). BES1 is activated by EMS1-TPD1-SERK1/2-mediated signaling to control tapetum development in Arabidopsis thaliana. Nat. Commun. 10, 4164. 10.1038/s41467-019-12118-4 31519953 PMC6744560

[B19] Chen X. (2008). MicroRNA metabolism in plants. Curr. Top. Microbiol. Immunol. 320, 117–136. 10.1007/978-3-540-75157-1_6 18268842 PMC2570777

[B20] Choi K. Zhao X. Tock A. J. Lambing C. Underwood C. J. Hardcastle T. J. (2018). Nucleosomes and DNA methylation shape meiotic DSB frequency in Arabidopsis thaliana transposons and gene regulatory regions. Genome Res. 28, 532–546. 10.1101/gr.225599.117 29530928 PMC5880243

[B21] Cnudde F. Hedatale V. de Jong H. Pierson E. S. Rainey D. Y. Zabeau M. (2006). Changes in gene expression during male meiosis in Petunia hybrida. Chromosome Res.: an Int. J. Mol. Supramol. Evol. Asp. Chromosome Biol. 14, 919–932. 10.1007/s10577-006-1099-5 17203374

[B22] Crismani W. Baumann U. Sutton T. Shirley N. Webster T. Spangenberg G. (2006). Microarray expression analysis of meiosis and microsporogenesis in hexaploid bread wheat. BMC Genomics 7, 267. 10.1186/1471-2164-7-267 17052357 PMC1647286

[B23] Cui Y. Hu C. Zhu Y. Cheng K. Li X. Wei Z. (2018). CIK receptor kinases determine cell fate specification during early anther development in Arabidopsis. Plant Cell 30, 2383–2401. 10.1105/tpc.17.00586 30201822 PMC6241272

[B24] De Storme N. Geelen D. (2013). Sexual polyploidization in plants–cytological mechanisms and molecular regulation. New Phytol. 198, 670–684. 10.1111/nph.12184 23421646 PMC3744767

[B25] Deveshwar P. Bovill W. D. Sharma R. Able J. A. Kapoor S. (2011). Analysis of anther transcriptomes to identify genes contributing to meiosis and male gametophyte development in rice. BMC Plant Biol. 11, 78. 10.1186/1471-2229-11-78 21554676 PMC3112077

[B26] Dhaka N. Sharma S. Vashisht I. Kandpal M. Sharma M. K. Sharma R. (2019). Small RNA profiling from meiotic and post-meiotic anthers reveals prospective miRNA-target modules for engineering male fertility in sorghum. Genomics. 10.1016/j.ygeno.2019.09.009 31521711

[B27] Dukowic-Schulze S. Harris A. Li J. Sundararajan A. Mudge J. Retzel E. F. (2014). Comparative transcriptomics of early meiosis in Arabidopsis and maize. J. Genet. Genomics 41, 139–152. 10.1016/j.jgg.2013.11.007 24656234

[B28] Dukowic-Schulze S. Sundararajan A. Ramaraj T. Kianian S. Pawlowski W. P. Mudge J. (2016). Novel meiotic miRNAs and indications for a role of PhasiRNAs in meiosis. Front. Plant Sci. 7, 762. 10.3389/fpls.2016.00762 27313591 PMC4889585

[B29] Fei Q. Xia R. Meyers B. C. (2013). Phased, secondary, small interfering RNAs in posttranscriptional regulatory networks. Plant Cell 25, 2400–2415. 10.1105/tpc.113.114652 23881411 PMC3753373

[B30] Fei Q. Yang L. Liang W. Zhang D. Meyers B. C. (2016). Dynamic changes of small RNAs in rice spikelet development reveal specialized reproductive phasiRNA pathways. J. Exp. Bot. 67, 6037–6049. 10.1093/jxb/erw361 27702997 PMC5100018

[B31] Feng B. M. Lu D. H. Ma X. Peng Y. B. Sun Y. J. Ning G. (2012). Regulation of the Arabidopsis anther transcriptome by DYT1 for pollen development. Plant J. 72, 612–624. 10.1111/j.1365-313X.2012.05104.x 22775442

[B32] Ferdous M. Higgins J. D. Osman K. Lambing C. Roitinger E. Mechtler K. (2012). Inter-homolog crossing-over and synapsis in Arabidopsis meiosis are dependent on the chromosome axis protein AtASY3. PloS Genet. 8, e1002507. 10.1371/journal.pgen.1002507 22319460 PMC3271061

[B33] Ferguson A. C. Pearce S. Band L. R. Yang C. Ferjentsikova I. King J. (2017). Biphasic regulation of the transcription factor ABORTED MICROSPORES (AMS) is essential for tapetum and pollen development in Arabidopsis. New Phytol. 213, 778–790. 10.1111/nph.14200 27787905 PMC5215365

[B34] Florez-Zapata N. M. Reyes-Valdes M. H. Hernandez-Godinez F. Martinez O. (2014). Transcriptomic landscape of prophase I sunflower male meiocytes. Front. Plant Sci. 5, 277. 10.3389/fpls.2014.00277 24982667 PMC4059168

[B35] Fu Z. Yu J. Cheng X. Zong X. Xu J. Chen M. (2014). The rice basic helix-loop-helix transcription factor TDR INTERACTING PROTEIN2 is a central switch in early anther development. Plant Cell 26, 1512–1524. 10.1105/tpc.114.123745 24755456 PMC4036568

[B36] Gao J. Li Q. Wang N. Tao B. Wen J. Yi B. (2019). Tapetal expression of BnaC.MAGL8.a causes male sterility in Arabidopsis. Front. Plant Sci. 10, 763–763. 10.3389/fpls.2019.00763 31249581 PMC6582705

[B37] Grelon M. Vezon D. Gendrot G. Pelletier G. (2001). AtSPO11-1 is necessary for efficient meiotic recombination in plants. EMBO J. 20, 589–600. 10.1093/emboj/20.3.589 11157765 PMC133473

[B38] Gu J. N. Zhu J. Yu Y. Teng X. D. Lou Y. Xu X. F. (2014). DYT1 directly regulates the expression of TDF1 for tapetum development and pollen wall formation in Arabidopsis. Plant J. 80, 1005–1013. 10.1111/tpj.12694 25284309

[B39] Guo W. J. Ho T. H. (2008). An abscisic acid-induced protein, HVA22, inhibits gibberellin-mediated programmed cell death in cereal aleurone cells. Plant Physiol. 147, 1710–1722. 10.1104/pp.108.120238 18583533 PMC2492636

[B40] Guo J. Liu C. Wang P. Cheng Q. Sun L. Yang W. (2018). The Aborted Microspores (AMS)-Like gene is required for anther and microspore development in pepper (Capsicum annuum L.). Int. J. Mol. Sci. 19. 10.3390/ijms19051341 PMC5983743 29724052

[B41] He Y. Wang M. Dukowic-Schulze S. Zhou A. Tiang C. L. Shilo S. (2017). Genomic features shaping the landscape of meiotic double-strand-break hotspots in maize. Proc. Natl. Acad. Sci. U.S.A. 114, 12231–12236. 10.1073/pnas.1713225114 29087335 PMC5699076

[B42] Higgins J. D. Sanchez-Moran E. Armstrong S. J. Jones G. H. Franklin F. C. (2005). The Arabidopsis synaptonemal complex protein ZYP1 is required for chromosome synapsis and normal fidelity of crossing over. Genes Dev. 19, 2488–2500. 10.1101/gad.354705 16230536 PMC1257403

[B43] Hiraguri A. Itoh R. Kondo N. Nomura Y. Aizawa D. Murai Y. (2005). Specific interactions between Dicer-like proteins and HYL1/DRB-family dsRNA-binding proteins in Arabidopsis thaliana. Plant Mol. Biol. 57, 173–188. 10.1007/s11103-004-6853-5 15821876

[B44] Huang J. Zhang T. Linstroth L. Tillman Z. Otegui M. S. Owen H. A. (2016). Control of anther cell differentiation by the small protein ligand TPD1 and its receptor EMS1 in Arabidopsis. PloS Genet. 12, e1006147. 10.1371/journal.pgen.1006147 27537183 PMC4990239

[B45] Huang J. Li Z. Biener G. Xiong E. Malik S. Eaton N. (2017). Carbonic anhydrases function in anther cell differentiation downstream of the receptor-like kinase EMS1. Plant Cell 29, 1335–1356. 10.1105/tpc.16.00484 28522549 PMC5502440

[B46] Huang J. Wang C. Wang H. Lu P. Zheng B. Ma H. (2019a). Meiocyte-specific and AtSPO11-1–dependent small RNAs and their association with meiotic gene expression and recombination. Plant Cell 31, 444–464. 10.1105/tpc.18.00511 30674694 PMC6447014

[B47] Huang K. Baldrich P. Meyers B. C. Caplan J. L. (2019b). sRNA-FISH: versatile fluorescent in situ detection of small RNAs in plants. Plant J. 98, 359–369. 10.1111/tpj.14210 30577085 PMC6465150

[B48] Ito T. Nagata N. Yoshiba Y. Ohme-Takagi M. Ma H. Shinozaki K. (2007). Arabidopsis MALE STERILITY1 encodes a PHD-type transcription factor and regulates pollen and tapetum development. Plant Cell 19, 3549–3562. 10.1105/tpc.107.054536 18032630 PMC2174881

[B49] Jeong H. J. Kang J. H. Zhao M. Kwon J. K. Choi H. S. Bae J. H. (2014). Tomato Male sterile 1035 is essential for pollen development and meiosis in anthers. J. Exp. Bot. 65, 6693–6709. 10.1093/jxb/eru389 25262227 PMC4246194

[B50] Ji C. Li H. Chen L. Xie M. Wang F. Chen Y. (2013). A novel rice bHLH transcription factor, DTD, acts coordinately with TDR in controlling tapetum function and pollen development. Mol. Plant 6, 1715–1718. 10.1093/mp/sst046 23519457

[B51] Jia G. X. Liu X. D. Owen H. A. Zhao D. Z. (2008). Signaling of cell fate determination by the TPD1 small protein and EMS1 receptor kinase. Proc. Natl. Acad. Sci. U.S.A. 105, 2220–2225. 10.1073/pnas.0708795105 18250314 PMC2538901

[B52] Jung K. H. Han M. J. Lee Y. S. Kim Y. W. Hwang I. Kim M. J. (2005). Rice Undeveloped Tapetum1 is a major regulator of early tapetum development. Plant Cell 17, 2705–2722. 10.1105/tpc.105.034090 16141453 PMC1242267

[B53] Kakrana A. Mathioni S. M. Huang K. Hammond R. Vandivier L. Patel P. (2018). Plant 24-nt reproductive phasiRNAs from intramolecular duplex mRNAs in diverse monocots. Genome Res. 28, 1333–1344. 10.1101/gr.228163.117 30002159 PMC6120631

[B54] Kamthan A. Chaudhuri A. Kamthan M. Datta A. (2015). Small RNAs in plants: recent development and application for crop improvement. Front. Plant Sci. 6, 208–208. 10.3389/fpls.2015.00208 25883599 PMC4382981

[B55] Kaneko M. Inukai Y. Ueguchi-Tanaka M. Itoh H. Izawa T. Kobayashi Y. (2004). Loss-of-function mutations of the rice GAMYB gene impair alpha-amylase expression in aleurone and flower development. Plant Cell 16, 33–44. 10.1105/tpc.017327 14688295 PMC301393

[B56] Ko S. S. Li M. J. Sun-Ben Ku M. Ho Y. C. Lin Y. J. Chuang M. H. (2014). The bHLH142 transcription factor coordinates with TDR1 to modulate the expression of EAT1 and regulate pollen development in rice. Plant Cell 26, 2486–2504. 10.1105/tpc.114.126292 24894043 PMC4114947

[B57] Komiya R. Ohyanagi H. Niihama M. Watanabe T. Nakano M. Kurata N. (2014). Rice germline-specific Argonaute MEL1 protein binds to phasiRNAs generated from more than 700 lincRNAs. Plant J. 78, 385–397. 10.1111/tpj.12483 24635777

[B58] Li N. Zhang D. S. Liu H. S. Yin C. S. Li X. X. Liang W. Q. (2006). The rice tapetum degeneration retardation gene is required for tapetum degradation and anther development. Plant Cell 18, 2999–3014. 10.1105/tpc.106.044107 17138695 PMC1693939

[B59] Li H. Yuan Z. Vizcay-Barrena G. Yang C. Liang W. Zong J. (2011). PERSISTENT TAPETAL CELL1 encodes a PHD-finger protein that is required for tapetal cell death and pollen development in rice. Plant Physiol. 156, 615–630. 10.1104/pp.111.175760 21515697 PMC3177263

[B60] Li D.-D. Xue J.-S. Zhu J. Yang Z.-N. (2017a). Gene regulatory network for tapetum development in Arabidopsis thaliana. Front. Plant Sci. 8, 1559–1559. 10.3389/fpls.2017.01559 28955355 PMC5601042

[B61] Li Z. Wang Y. Huang J. Ahsan N. Biener G. Paprocki J. (2017b). Two SERK receptor-like kinases interact with EMS1 to control anther cell fate determination. Plant Physiol. 173, 326–337. 10.1104/pp.16.01219 27920157 PMC5210720

[B62] Liu B. Chen Z. Song X. Liu C. Cui X. Zhao X. (2007). Oryza sativa dicer-like4 reveals a key role for small interfering RNA silencing in plant development. Plant Cell 19, 2705–2718. 10.1105/tpc.107.052209 17905898 PMC2048709

[B63] Liu Z. Bao W. Liang W. Yin J. Zhang D. (2010). Identification of gamyb-4 and analysis of the regulatory role of GAMYB in rice anther development. J. Integr. Plant Biol. 52, 670–678. 10.1111/j.1744-7909.2010.00959.x 20590996

[B64] Liu B. De Storme N. Geelen D. (2017). Gibberellin induces diploid pollen formation by interfering with meiotic cytokinesis. Plant Physiol. 173, 338–353. 10.1104/pp.16.00480 27621423 PMC5210705

[B65] Lou Y. Zhou H. S. Han Y. Zeng Q. Y. Zhu J. Yang Z. N. (2018). Positive regulation of AMS by TDF1 and the formation of a TDF1-AMS complex are required for anther development in Arabidopsis thaliana. New Phytol. 217, 378–391. 10.1111/nph.14790 28940573

[B66] Lu P. Chai M. Yang J. Ning G. Wang G. Ma H. (2014). The Arabidopsis CALLOSE DEFECTIVE MICROSPORE1 gene is required for male fertility through regulating callose metabolism during microsporogenesis. Plant Physiol. 164, 1893–1904. 10.1104/pp.113.233387 24567187 PMC3982751

[B67] Ma X. Feng B. Ma H. (2012). AMS-dependent and independent regulation of anther transcriptome and comparison with those affected by other Arabidopsis anther genes. BMC Plant Biol. 12, 23. 10.1186/1471-2229-12-23 22336428 PMC3305669

[B68] Mercier R. Mézard C. Jenczewski E. Macaisne N. Grelon M. (2015). The molecular biology of meiosis in plants. Annu. Rev. Plant Biol. 66, 297–327. 10.1146/annurev-arplant-050213-035923 25494464

[B69] Millar A. A. Gubler F. (2005). The Arabidopsis GAMYB-like genes, MYB33 and MYB65, are microRNA-regulated genes that redundantly facilitate anther development. Plant Cell 17, 705–721. 10.1105/tpc.104.027920 15722475 PMC1069693

[B70] Mondol P. C. Xu D. Duan L. Shi J. Wang C. Chen X. (2019). Defective Pollen Wall 3 (DPW3), a novel alpha integrin-like protein, is required for pollen wall formation in rice. New Phytol. 10.1111/nph.16161 31486533

[B71] Murmu J. Bush M. J. DeLong C. Li S. Xu M. Khan M. (2010). Arabidopsis basic leucine-zipper transcription factors TGA9 and TGA10 interact with floral glutaredoxins ROXY1 and ROXY2 and are redundantly required for anther development. Plant Physiol. 154, 1492–1504. 10.1104/pp.110.159111 20805327 PMC2971623

[B72] Niu N. Liang W. Yang X. Jin W. Wilson Z. A. Hu J. (2013). EAT1 promotes tapetal cell death by regulating aspartic proteases during male reproductive development in rice. Nat. Commun. 4, 1445. 10.1038/ncomms2396 23385589

[B73] Nonomura K. Miyoshi K. Eiguchi M. Suzuki T. Miyao A. Hirochika H. (2003). The MSP1 gene is necessary to restrict the number of cells entering into male and female sporogenesis and to initiate anther wall formation in rice. Plant Cell 15, 1728–1739. 10.1105/tpc.012401 12897248 PMC167165

[B74] Nonomura K. Morohoshi A. Nakano M. Eiguchi M. Miyao A. Hirochika H. (2007). A germ cell specific gene of the ARGONAUTE family is essential for the progression of premeiotic mitosis and meiosis during sporogenesis in rice. Plant Cell 19, 2583–2594. 10.1105/tpc.107.053199 17675402 PMC2002623

[B75] Oliver C. Pradillo M. Jover-Gil S. Cunado N. Ponce M. R. Santos J. L. (2017). Loss of function of Arabidopsis microRNA-machinery genes impairs fertility, and has effects on homologous recombination and meiotic chromatin dynamics. Sci. Rep. 7, 9280. 10.1038/s41598-017-07702-x 28839139 PMC5571030

[B76] Omidvar V. Mohorianu I. Dalmay T. Fellner M. (2015). Identification of miRNAs with potential roles in regulation of anther development and male-sterility in 7B-1 male-sterile tomato mutant. BMC Genomics 16, 878–878. 10.1186/s12864-015-2077-0 26511108 PMC4625851

[B77] Ono S. Liu H. Tsuda K. Fukai E. Tanaka K. Sasaki T. (2018). EAT1 transcription factor, a non-cell-autonomous regulator of pollen production, activates meiotic small RNA biogenesis in rice anther tapetum. PloS Genet. 14, e1007238. 10.1371/journal.pgen.1007238 29432414 PMC5825165

[B78] Osman K. Yang J. Roitinger E. Lambing C. Heckmann S. Howell E. (2018). Affinity proteomics reveals extensive phosphorylation of the Brassica chromosome axis protein ASY1 and a network of associated proteins at prophase I of meiosis. Plant J. 93, 17–33. 10.1111/tpj.13752 29078019 PMC5767750

[B79] Park W. Li J. Song R. Messing J. Chen X. (2002). CARPEL FACTORY, a Dicer homolog, and HEN1, a novel protein, act in microRNA metabolism in Arabidopsis thaliana. Curr. Biol.: CB 12, 1484–1495. 10.1016/s0960-9822(02)01017-5 12225663 PMC5137372

[B80] Park M. Y. Wu G. Gonzalez-Sulser A. Vaucheret H. Poethig R. S. (2005). Nuclear processing and export of microRNAs in Arabidopsis. P. Natl. Acad. Sci. U.S.A. 102, 3691–3696. 10.1073/pnas.0405570102 PMC553294 15738428

[B81] Patel P. Mathioni S. Kakrana A. Shatkay H. Meyers B. C. (2018). Reproductive phasiRNAs in grasses are compositionally distinct from other classes of small RNAs. New Phytol. 220, 851–864. 10.1111/nph.15349 30020552

[B82] Peng J. Carol P. Richards D. E. King K. E. Cowling R. J. Murphy G. P. (1997). The Arabidopsis GAI gene defines a signaling pathway that negatively regulates gibberellin responses. Genes Dev. 11, 3194–3205. 10.1101/gad.11.23.3194 9389651 PMC316750

[B83] Plackett A. R. G. Thomas S. G. Wilson Z. A. Hedden P. (2011). Gibberellin control of stamen development: a fertile field. Trends Plant Sci. 16, 568–578. 10.1016/j.tplants.2011.06.007 21824801

[B84] Plackett A. R. Ferguson A. C. Powers S. J. Wanchoo-Kohli A. Phillips A. L. Wilson Z. A. (2014). DELLA activity is required for successful pollen development in the Columbia ecotype of Arabidopsis. New Phytol. 201, 825–836. 10.1111/nph.12571 24400898 PMC4291109

[B85] Qi Y. He X. Wang X.-J. Kohany O. Jurka J. Hannon G. J. (2006). Distinct catalytic and non-catalytic roles of ARGONAUTE4 in RNA-directed DNA methylation. Nature 443, 1008–1012. 10.1038/nature05198 16998468

[B86] Reinhart B. J. Weinstein E. G. Rhoades M. W. Bartel B. Bartel D. P. (2002). MicroRNAs in plants. Genes Dev. 16, 1616–1626. 10.1101/gad.1004402 12101121 PMC186362

[B87] Sanders P. M. Bui A. Q. Weterings K. McIntire K. N. Hsu Y.-C. Lee P. Y. (1999). Anther developmental defects in Arabidopsis thaliana male-sterile mutants. Plant Reprod. 11, 297–322. 10.1007/s004970050158

[B88] Schommer C. Beven A. Lawrenson T. Shaw P. Sablowski R. (2003). AHP2 is required for bivalent formation and for segregation of homologous chromosomes in Arabidopsis meiosis. Plant J. 36, 1–11. 10.1046/j.1365-313x.2003.01850.x 12974806

[B89] Scott R. J. Spielman M. Dickinson H. G. (2004). Stamen structure and function. Plant Cell 16 Suppl, S46–S60. 10.1105/tpc.017012 15131249 PMC2643399

[B90] Shukla P. Gautam R. Singh N. K. Ahmed I. Kirti P. B. (2019). A proteomic study of cysteine protease induced cell death in anthers of male sterile tobacco transgenic plants. Physiol. Mol. Biol. Plants: Int. J. Funct. Plant Biol. 25, 1073–1082. 10.1007/s12298-019-00642-y PMC6656835 31402825

[B91] Silverstone A. L. Mak P. Y. Martínez E. C. Sun T. P. (1997). The new RGA locus encodes a negative regulator of gibberellin response in Arabidopsis thaliana. Genetics 146, 1087–1099. 9215910 10.1093/genetics/146.3.1087 PMC1208037

[B92] Silverstone A. L. Ciampaglio C. N. Sun T.-p. (1998). The Arabidopsis RGA gene encodes a transcriptional regulator repressing the gibberellin signal transduction pathway. Plant Cell 10, 155–169. 10.1105/tpc.10.2.155 9490740 PMC143987

[B93] Song X. Li P. Zhai J. Zhou M. Ma L. Liu B. (2012). Roles of DCL4 and DCL3b in rice phased small RNA biogenesis. Plant J. 69, 462–474. 10.1111/j.1365-313X.2011.04805.x 21973320

[B94] Sun L. Sun G. Shi C. Sun D. (2018a). Transcriptome analysis reveals new microRNAs-mediated pathway involved in anther development in male sterile wheat. BMC Genomics 19, 333. 10.3390/ijms19124017 29739311 PMC5941544

[B95] Sun L. Xiang X. Yang Z. Yu P. Wen X. Wang H. (2018b). OsGPAT3 plays a critical role in anther wall programmed cell death and pollen development in rice. Int. J. Mol. Sci. 19. 10.3390/ijms19124017 PMC6321289 30545137

[B96] Sun W. Xiang X. Zhai L. Zhang D. Cao Z. Liu L. (2018c). AGO18b negatively regulates determinacy of spikelet meristems on the tassel central spike in maize. J. Integr. Plant Biol. 60, 65–78. 10.1111/jipb.12596 28875539

[B97] Sun W. Chen D. Xue Y. Zhai L. Zhang D. Cao Z. (2019). Genome-wide identification of AGO18b-bound miRNAs and phasiRNAs in maize by cRIP-seq. BMC Genomics 20, 656. 10.1186/s12864-019-6028-z 31419938 PMC6697968

[B98] Tamim S. Cai Z. Mathioni S. M. Zhai J. Teng C. Zhang Q. (2018). Cis-directed cleavage and nonstoichiometric abundances of 21-nucleotide reproductive phased small interfering RNAs in grasses. New Phytol. 220, 865–877. 10.1111/nph.15181 29708601

[B99] Teng C. Zhang H. Hammond R. Huang K. Meyers B. C. Walbot V. (2018). Dicer-like 5 deficiency confers temperature-sensitive male sterility in maize. BioRxiv. 498410. 10.1101/498410 PMC7283321 32518237

[B100] Underwood C. J. Choi K. Lambing C. Zhao X. Serra H. Borges F. (2018). Epigenetic activation of meiotic recombination near Arabidopsis thaliana centromeres via loss of H3K9me2 and non-CG DNA methylation. Genome Res. 28, 519–531. 10.1101/gr.227116.117 29530927 PMC5880242

[B101] Verma N. (2019). Transcriptional regulation of anther development in Arabidopsis. Gene 689, 202–209. 10.1016/j.gene.2018.12.022 30572098

[B102] Walker J. Gao H. Zhang J. Aldridge B. Vickers M. Higgins J. D. (2018). Sexual-lineage-specific DNA methylation regulates meiosis in Arabidopsis. Nat. Genet. 50, 130–137. 10.1038/s41588-017-0008-5 29255257 PMC7611288

[B103] Wang Y. Copenhaver G. P. (2018). Meiotic recombination: mixing it up in plants. Annu. Rev. Plant Biol. 69, 577–609. 10.1146/annurev-arplant-042817-040431 29489392

[B104] Wang K. Wang M. Tang D. Shen Y. Qin B. Li M. (2011). PAIR3, an axis-associated protein, is essential for the recruitment of recombination elements onto meiotic chromosomes in rice. Mol. Biol. Cell 22, 12–19. 10.1091/mbc.E10-08-0667 21119003 PMC3016970

[B105] Wang C. J. Nan G. L. Kelliher T. Timofejeva L. Vernoud V. Golubovskaya I. N. (2012). Maize multiple archesporial cells 1 (mac1), an ortholog of rice TDL1A, modulates cell proliferation and identity in early anther development. Development 139, 2594–2603. 10.1242/dev.077891 22696296 PMC4496874

[B106] Wang J. Niu B. Huang J. Wang H. Yang X. Dong A. (2016). The PHD finger protein MMD1/DUET ensures the progression of male meiotic chromosome condensation and directly regulates the expression of the condensin gene CAP-D3. Plant Cell 28, 1894–1909. 10.1105/tpc.16.00040 27385818 PMC5006699

[B107] Xia R. Chen C. Pokhrel S. Ma W. Huang K. Patel P. (2019). 24-nt reproductive phasiRNAs are broadly present in angiosperms. Nat. Commun. 10, 627. 10.1038/s41467-019-08543-0 30733503 PMC6367383

[B108] Xu J. Yang C. Yuan Z. Zhang D. Gondwe M. Y. Ding Z. (2010). The ABORTED MICROSPORES regulatory network is required for postmeiotic male reproductive development in Arabidopsis thaliana. Plant Cell 22, 91–107. 10.1105/tpc.109.071803 20118226 PMC2828693

[B109] Xu D. Qu S. Tucker M. R. Zhang D. Liang W. Shi J. (2019). Ostkpr1 functions in anther cuticle development and pollen wall formation in rice. BMC Plant Biol. 19, 104. 10.1186/s12870-019-1711-4 30885140 PMC6421701

[B110] Xue M. Wang J. Jiang L. Wang M. Wolfe S. Pawlowski W. P. (2018). The number of meiotic double-strand breaks influences crossover distribution in Arabidopsis. Plant Cell 30, 2628–2638. 10.1105/tpc.18.00531 30282794 PMC6241269

[B111] Yang W.-C. Ye D. Xu J. Sundaresan V. (1999). The SPOROCYTELESS gene of Arabidopsis is required for initiation of sporogenesis and encodes a novel nuclear protein. Genes Dev. 13, 2108–2117. 10.1101/gad.13.16.2108 10465788 PMC316961

[B112] Yang S. L. Xie L. F. Mao H. Z. Puah C. S. Yang W. C. Jiang L. (2003). Tapetum determinant1 is required for cell specialization in the Arabidopsis anther. Plant Cell 15, 2792–2804. 10.1105/tpc.016618 14615601 PMC282801

[B113] Yang S. L. Jiang L. Puah C. S. Xie L. F. Zhang X. Q. Chen L. Q. (2005). Overexpression of TAPETUM DETERMINANT1 alters the cell fates in the Arabidopsis carpel and tapetum via genetic interaction with excess microsporocytes1/extra sporogenous cells. Plant Physiol. 139, 186–191. 10.1104/pp.105.063529 16055681 PMC1203368

[B114] Yang C. Vizcay-Barrena G. Conner K. Wilson Z. A. (2007). MALE STERILITY1 is required for tapetal development and pollen wall biosynthesis. Plant Cell 19, 3530–3548. 10.1105/tpc.107.054981 18032629 PMC2174882

[B115] Yang H. Lu P. Wang Y. Ma H. (2011). The transcriptome landscape of Arabidopsis male meiocytes from high-throughput sequencing: the complexity and evolution of the meiotic process. Plant J. 65, 503–516. 10.1111/j.1365-313X.2010.04439.x 21208307

[B116] Yang L. Qian X. Chen M. Fei Q. Meyers B. C. Liang W. (2016). Regulatory role of a receptor-like kinase in specifying anther cell identity. Plant Physiol. 171, 2085–2100. 10.1104/pp.16.00016 27208278 PMC4936546

[B117] Yang C. Hamamura Y. Sofroni K. Böwer F. Stolze S. C. Nakagami H. (2019a). SWITCH 1/DYAD is a WINGS APART-LIKE antagonist that maintains sister chromatid cohesion in meiosis. Nat. Commun. 10, 1755–1755. 10.1038/s41467-019-09759-w 30988453 PMC6465247

[B118] Yang C. Sofroni K. Wijnker E. Hamamura Y. Carstens L. Harashima H. (2019b). The Arabidopsis Cdk1/Cdk2 homolog CDKA;1 controls chromosome axis assembly during plant meiosis. EMBO J., e101625. 10.15252/embj.2019101625 31556459 PMC6996576

[B119] Yang Z. Liu L. Sun L. Yu P. Zhang P. Abbas A. (2019c). OsMS1 functions as a transcriptional activator to regulate programmed tapetum development and pollen exine formation in rice. Plant Mol. Biol. 99, 175–191. 10.1007/s11103-018-0811-0 30610522

[B120] Yang Z. Sun L. Zhang P. Zhang Y. Yu P. Liu L. (2019d). TDR INTERACTING PROTEIN 3, encoding a PHD-finger transcription factor, regulates Ubisch bodies and pollen wall formation in rice. Plant J. 99, 844–861. 10.1111/tpj.14365 31021015 PMC6852570

[B121] Yelina N. E. Lambing C. Hardcastle T. J. Zhao X. Santos B. Henderson I. R. (2015). DNA methylation epigenetically silences crossover hot spots and controls chromosomal domains of meiotic recombination in Arabidopsis. Genes Dev. 29, 2183–2202. 10.1101/gad.270876.115 26494791 PMC4617981

[B122] Yi J. Kim S. R. Lee D. Y. Moon S. Lee Y. S. Jung K. H. (2012). The rice gene DEFECTIVE TAPETUM AND MEIOCYTES 1 (DTM1) is required for early tapetum development and meiosis. Plant J. 70, 256–270. 10.1111/j.1365-313X.2011.04864.x 22111585

[B123] Yi J. Moon S. Lee Y. S. Zhu L. Liang W. Zhang D. (2016). Defective Tapetum Cell Death 1 (DTC1) regulates ROS levels by binding to metallothionein during tapetum degeneration. Plant Physiol. 170, 1611–1623. 10.1104/pp.15.01561 26697896 PMC4775127

[B124] Yin Y. Vafeados D. Tao Y. Yoshida S. Asami T. Chory J. (2005). A new class of transcription factors mediates brassinosteroid-regulated gene expression in Arabidopsis. Cell 120, 249–259. 10.1016/j.cell.2004.11.044 15680330

[B125] Yu B. Yang Z. Li J. Minakhina S. Yang M. Padgett R. W. (2005). Methylation as a crucial step in plant microRNA biogenesis. Science 307, 932–935. 10.1126/science.1107130 15705854 PMC5137370

[B126] Yuan G. Ahootapeh B. H. Komaki S. Schnittger A. Lillo C. De Storme N. (2018). PROTEIN PHOSHATASE 2A B’alpha and beta Maintain Centromeric Sister Chromatid Cohesion during Meiosis in Arabidopsis. Plant Physiol. 178, 317–328. 10.1104/pp.18.00281 30061120 PMC6130024

[B127] Zhai L. Sun W. Zhang K. Jia H. Liu L. Liu Z. (2014). Identification and characterization of Argonaute gene family and meiosis-enriched Argonaute during sporogenesis in maize. J. Integr. Plant Biol. 56, 1042–1052. 10.1111/jipb.12205 24735215

[B128] Zhai J. Zhang H. Arikit S. Huang K. Nan G. L. Walbot V. (2015). Spatiotemporally dynamic, cell-type-dependent premeiotic and meiotic phasiRNAs in maize anthers. Proc. Natl. Acad. Sci. U.S.A. 112, 3146–3151. 10.1073/pnas.1418918112 25713378 PMC4364226

[B129] Zhang W. Sun Y. Timofejeva L. Chen C. Grossniklaus U. Ma H. (2006). Regulation of Arabidopsis tapetum development and function by DYSFUNCTIONAL TAPETUM1 (DYT1) encoding a putative bHLH transcription factor. Development 133, 3085–3095. 10.1242/dev.02463 16831835

[B130] Zhang Z.-B. Zhu J. Gao J.-F. Wang C. Li H. Li H. (2007). Transcription factor AtMYB103 is required for anther development by regulating tapetum development, callose dissolution and exine formation in Arabidopsis. Plant J. 52, 528–538. 10.1111/j.1365-313X.2007.03254.x 17727613

[B131] Zhang D. S. Liang W. Q. Yuan Z. Li N. Shi J. Wang J. (2008). Tapetum degeneration retardation is critical for aliphatic metabolism and gene regulation during rice pollen development. Mol. Plant 1, 599–610. 10.1093/mp/ssn028 19825565

[B132] Zhang S. Fang Z. Zhu J. Gao J. Yang Z. (2010). OsMYB103 is required for rice anther development by regulating tapetum development and exine formation. Chin. Sci. Bull. 55, 3288–3297. 10.1007/s11434-010-4087-2

[B133] Zhang Y. L. Zhang H. Gao Y. J. Yan L. L. Yu X. Y. Yang Y. H. (2019). Protein Phosphatase 2A B’alpha and B’beta protect centromeric cohesion during Meiosis I. Plant Physiol. 179, 1556–1568. 10.1104/pp.18.01320 30705069 PMC6446778

[B134] Zhao D. Z. Wang G. F. Speal B. Ma H. (2002). The excess microsporocytes1 gene encodes a putative leucine-rich repeat receptor protein kinase that controls somatic and reproductive cell fates in the Arabidopsis anther. Genes Dev. 16, 2021–2031. 10.1101/gad.997902 12154130 PMC186413

[B135] Zheng Y. Wang Y. Wu J. Ding B. Fei Z. (2015). A dynamic evolutionary and functional landscape of plant phased small interfering RNAs. BMC Biol. 13, 32. 10.1186/s12915-015-0142-4 25980406 PMC4457045

[B136] Zheng S. Li J. Ma L. Wang H. Zhou H. Ni E. (2019). OsAGO2 controls ROS production and the initiation of tapetal PCD by epigenetically regulating OsHXK1 expression in rice anthers. Proc. Natl. Acad. Sci. U.S.A. 116, 7549–7558. 10.1073/pnas.1817675116 30902896 PMC6462063

[B137] Zhou S. Wang Y. Li W. Zhao Z. Ren Y. Wang Y. (2011). Pollen semi-sterility1 encodes a kinesin-1-like protein important for male meiosis, anther dehiscence, and fertility in rice. Plant Cell 23, 111–129. 10.1105/tpc.109.073692 21282525 PMC3051251

